# Dynamic modulation of the perceptual load on microsaccades during a selective spatial attention task

**DOI:** 10.1038/s41598-017-16629-2

**Published:** 2017-11-28

**Authors:** Linyan Xue, Dan Huang, Tong Wang, Qiyi Hu, Xinyu Chai, Liming Li, Yao Chen

**Affiliations:** 1 School of Biomedical Engineering, Shanghai Jiao Tong University, Shanghai, 200240 China; 2 School of Quality and Technical Supervision, Hebei University, Baoding, 071002 China

## Abstract

Selective spatial attention enhances task performance at restricted regions within the visual field. The magnitude of this effect depends on the level of attentional load, which determines the efficiency of distractor rejection. Mechanisms of attentional load include perceptual selection and/or cognitive control involving working memory. Recent studies have provided evidence that microsaccades are influenced by spatial attention. Therefore, microsaccade activities may be exploited to help understand the dynamic control of selective attention under different load levels. However, previous reports in humans on the effect of attentional load on microsaccades are inconsistent, and it is not clear to what extent these results and the dynamic changes of microsaccade activities are similar in monkeys. We trained monkeys to perform a color detection task in which the perceptual load was manipulated by task difficulty with limited involvement of working memory. Our results indicate that during the task with high perceptual load, the rate and amplitude of microsaccades immediately before the target color change were significantly suppressed. We also found that the occurrence of microsaccades before the monkeys’ detection response deteriorated their performance, especially in the hard task. We propose that the activity of microsaccades might be an efficacious indicator of the perceptual load.

## Introduction

Duing gaze fixation, our eyes are not still but often perform small and involuntary movements. There are three distinct types of fixational eye movements: drift, tremor and microsaccades. Among them, microsaccades represent the fastest component, with amplitudes of generally less than 1°^1–3^. Microsaccades and saccades have similar properties and may share the common mechanisms that are involved in orienting attention in space and time^4,5^. One intriguing line of research has established a tight link between covert spatial attention and some microsaccade activities. The microsaccade rate sharply dropped after the onset of an attentional cue, followed by a temporary enhancement^6,7^, and their direction was shown to be modulated by a shift in attention focus induced either endogenously^8–10^ or exogenously^11,12^. On the other hand, a more recent study revealed that the direction of spontaneous microsaccades inherently reflected the shift of spatial attention^13^. All these findings suggest that microsaccades are closely correlated with selective spatial attention and could reveal the deployment of attention focus. Therefore, microsaccades could be used as a psychophysiological measurement of cognition^8,14^.

Selective attention facilitates the perception of stimuli while ignoring or suppressing task-irrelevant distractors. The magnitude of this attention-mediated enhancement depends on the attentional load, which could be defined as either the number of perceived items or the complexity of the perceptual identification^15,16^. Mechanisms of the attentional load have been proposed as perceptual load, depending on the perceptual demands of the stimulus, and/or cognitive load, traditionally depending on the higher demands of working memory^17^. The load theory^17–19^ suggests that increasing the perceptual load decreases distractor interference, whereas increasing the working memory load enhances distractor competition. These suggestions have been verified by neuroimaging studies^20–22^.

In humans, it was reported that microsaccade activities were influenced by the attentional load in visual tasks^9,23,24^ or nonvisual cognitive tasks^25–29^, such as arithmetic operation and digit retention, in which the primary mental process does not rely on vision. However, previous research investigating the relationship between the attentional load and the microsaccade rate obtained inconsistent results. Some studies have indicated that tasks with higher attentional load lead to a lower microsaccade rate. For example, Pastukhov and Braun^9^ used visual recognition tasks that required either a low attentional load (reporting color) or a high attentional load (reporting letter shape). They found a lower microsaccade rate and better microsaccade directional congruency associated with higher attentional load. Siegenthaler *et al*.^27^ required the human subjects to mentally count forward (low load) or backward (high load) in a mental arithmetic task and showed that the microsaccade rate decreased and the microsaccade amplitude increased, corresponding to higher working memory load. Gao *et al*.^28^ employed another mental arithmetic task in which the second operand was either small (low load) or large (high load). They also revealed an inverse relationship between the microsaccade rate and task difficulty, and reported that the extent of microsaccade rate inhibition was related to the task difficulty. More recently, Dalmaso *et al*.^29^ investigated the association between the working memory load and the microsaccade rate in two-digit (low load) or five-digit (high load) number memorizing tasks. Their results revealed that the microsaccade rate was significantly suppressed in the task with high working memory load. In contrast, other studies have revealed a positive correlation between the microsaccade rate and the task demand. For example, Benedetto *et al*.^23^ conducted simulated driving tasks either under low load (control task) or high load (dual task including visual search), and significantly more microsaccades were observed under the latter condition. Hicheur *et al*.^24^ employed a forced choice-task paradigm in which participants had to judge the orientation of a titled stimulus embedded in static or dynamic backgrounds. They found significantly higher microsaccade rate when participants were engaged in the execution of the discrimination task (high load) compared to that when they did not need to make response (low load). Besides, it should be noticed that these studies also showed the discrepancy in the effect of attentional load on other microsaccade parameters, such as the microsaccade amplitude.

These inconsistencies in the correlation between the attentional load and microsaccades could be attributed to the complexity of their tasks. Therefore, there could be complicated interactions between the effects of the perceptual and the working memory load. To avoid the influence of working memory, a task primarily with the perceptual load manipulated should be conducted. The use of awake, fixating monkeys has significant advantages to investigating neuronal mechanisms of microsaccades and their relationship to attentional load. Even though microsaccades have been shown to be modulated by different levels of cognitive process in humans, it is not known to what extent these results are similar in monkeys. Moreover, clarification is needed of the dynamic link between the perceptual load and microsaccades, which in turn would offer a unique window probing the attentional load.

The present study aimed to investigate whether microsaccade behaviors could be modulated by the perceptual load while monkeys performed a selective spatial attention task. For this purpose, we designed a demanding color detection task in which the attention focus was oriented by a previously presented cue. Task difficulty was manipulated by the noticeability of the color change in order to yield different levels of the perceptual load. Two features make our paradigm suitable for measuring cue- and stimulus-related microsaccade responses influenced by the perceptual load. The first is that our stimuli were kept identical before response events across different conditions, which can eliminate the influence of the stimulus variation under task-difficulty modulation. The second feature is that the detection task could be achieved with limited involvement of working memory. We found that the microsaccade rate immediately before the target color change was significantly suppressed while the perceptual load increased. Specifically, the microsaccade amplitude and its fluctuation were inhibited by the perceptual load during all the periods after the cue onset. Our results indicate that the microsaccade rate and amplitude might reveal the level of the perceptual load. We propose that microsaccades could be used as an indicator of the perceptual load level.

## Materials and methods

### Animal preparation and surgical procedures

We collected data from two (monkey G and monkey N) adult male rhesus monkeys (Macaca mulatta) that were 8 years of age and weighed 7–9.5 kg. All the animal procedures were approved by the Institutional Animal Care and Use Committee of Shanghai Jiao Tong University and followed the recommendations of the National Institutes of Health Guide for the Care and Use of Laboratory Animals.

Similar to our previous surgical procedures^30^, each macaque was implanted with a titanium head-post for head stabilization and a single sclera eye coil to monitor eye movements. During the behavioral training, the animals had controlled water access and obtained most of their fluid intake (water or juice) by performing the behavioral tasks.

### Apparatus

The stimulus patterns were generated by a computer running Visionworks (Vision Research Graphics, Durham, USA) and were presented on a gamma-corrected CRT monitor (GDM-F520 Monitor, Sony Electronics, Tokyo, Japan, 640 × 480 pixels, 150 Hz refresh rate) positioned 57 cm from the monkey’s eyes. The mean luminance of the screen was 22 cd/m^2^. Another computer running CORTEX (NIMH Laboratory of Neuropsychology) was used to control the different events of the behavioral task (reward time and amount, maintenance of visual fixation, bar touch, bar release and time of stimulus presentation). A computer running OmniPlex (Plexon Corp., Dallas, USA) was used for data acquisition. The reward system was controlled by a peristaltic pump (BT100M, Chuangrui, Baoding, China). Eye movements were measured by the eye tracking system (ScleraTrak, Crist Instrument, Maryland, USA) using the magnetic induction technique^31^ and sampling at 1 kHz.

### Behavioral tasks

**Figure 1:**
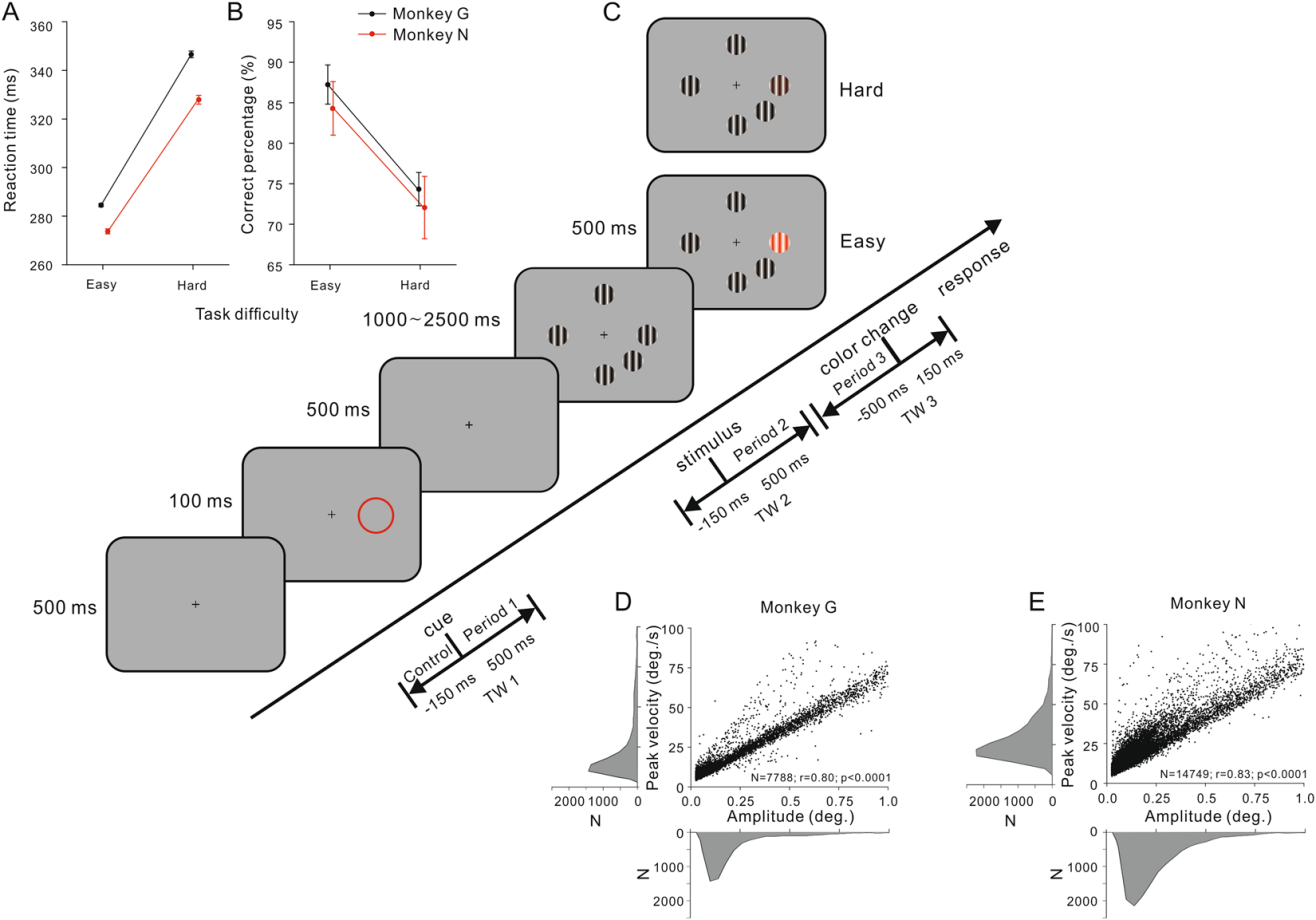
Task paradigm, behavioral performance and main sequences. (**A**,**B**) Changes in RT (left column) and response accuracy (right column) while performing tasks under different difficulties by monkey G (black) and monkey N (red). (**C**) Illustration of the experimental paradigm. Two rhesus monkeys were trained to fixate on a small cross while covertly attending to a spatial location that was cued 100 ms after the beginning of each trial. The cue was a thin red ring. After the cue ring disappeared for 500 ms, five drifting gratings were presented at iso-eccentricity locations. The grating that appeared at the cued position changed color after a random interval, and the animal was tasked with detecting the change by releasing a bar within 500 ms. The perceptual load modulations on microsaccades were measured in three different time windows (TWs) and four time periods. The Control period (150 ms before the cue onset) was defined as the baseline of microsaccade activities. The others were defined as 500 ms after the onsets of the cue and the stimulus and 500 ms preceding the color change. (**D**,**E**) Main sequences. Main panels: Peak velocity as a function of the microsaccade amplitude, including all microsaccades of monkey G and N. Each black dot corresponds to a single microsaccade. Bottom and side panels: Distributions of the microsaccade amplitude and peak velocity.

Microsaccade modulations by the spatial attention and the perceptual load were measured while the monkeys carried out a selective spatial attention task, which consisted of two detection conditions (easy and hard) based on the animals’ behavioral performance. As shown in Fig. 1C, the monkeys sitting in their training chairs were instructed to fixate on a white cross with a diameter of 0.1° at the center of the screen, which was presented throughout the entire experiment. When the monkey brought its gaze into a window of ±1° around the fixation point for 500 ms, a red ring with a diameter of 3° appeared for 100 ms and served as a cue to indicate the spatial location to be attended to. Within each task block, the ring was randomly shown at one of five different locations, which were equidistant from the point of fixation (8.8°). After the ring disappeared for 500 ms, five drifting gratings were presented at the aforementioned positions. They were sinusoidal gratings with a contrast of 100%, a diameter of 2°, a spatial frequency of 1 cycle/degree and a temporal frequency of 2 Hz. One of the five gratings was inside the cued position, whereas the others were outside. Following a randomized period of time (1–2.5 s), the grating at the cued location changed color, and the monkey was trained to release the bar as fast as possible to obtain a reward. The grating color was changed at its trough and could be either very noticeable (easy task, Look-up Table: 90–120, 0, 0) or difficult to detect (hard task, Look-up Table: 50–80, 0, 0). Once the grating changed color, the monkey had 500 ms to release the bar and received a reward. A shorter reaction time (RT) would result in a larger amount of reward. In both tasks, the animals were highly motivated to obtain more rewards. Because the animal would fail to receive a reward if the RTs were longer than 500 ms or shorter than 100 ms, it had to make greater effort during the hard task than during the easy task to obtain equal rewards. The color change was adjusted at each recording date to ensure the RTs during the hard task were significantly longer than those during the easy task (*p* < 0.05, analysis of variance, ANOVA).

### Microsaccade detection

Monocular microsaccades were detected from the onset of the fixation cross to 150 ms after the color change using an adaptation of a previously described algorithm developed by Engbert and Mergenthaler^32^. The time series of eye positions was transformed into velocities calculated over a moving window of twelve data points. Microsaccades were defined as periods during which the following conditions were satisfied: (1) the angular eye velocity exceeded a combined threshold for vertical and horizontal components equal to six times the median-based standard deviation of the velocity distribution (λ = 6); (2) since trials were automatically aborted when the monkey moved the eyes outside of the fixation window (1°), there were no microsaccade amplitude larger than 1° in the correct trials, the amplitude was set between 0.03° (2 arc min) and 1°^33^; (3) the minimum microsaccadic duration was set to 6 ms^33,34^; and (4) the maximum peak velocity was set to 100 °/s to keep the detected microsaccades close to the main sequence. Additionally, any microsaccades detected in the 20 ms following the end of the previous one was merged to exclude potential overshoot corrections^35^ and was excluded from the following analysis. To further verify the validity, we visually inspected each individual microsaccade. After all of these steps, we examined whether each microsaccade satisfied the main sequence criterion^36^. The significant linear relationship between the amplitudes and and their respective peak velocities (main sequence) is depicted in the lower right of Fig. 1. Corresponding distributions of the microsaccade amplitude and peak velocity are shown in the bottom and side panels (see Fig. 1D,E). Moreover, we compared the properties of microsaccades for our experiments to previous studies on microsaccades in non-human primates (Table 1 in Martinez-Conde *et al*.)^37^. The microsaccade parameters from our experiments were consistent with those previously reported.

**Table 1:** Comparison of the amplitudes of the Fano factors between the easy (first two rows) and hard (second two rows) tasks during Periods 1, 2 and 3 for monkeys G (first three columns) and N (last three columns).

	monkey G			monkey N		
	Period 1	Period 2	Period 3	Period 1	Period 2	Period 3
Fano factor	Easy: 0.302 ± 0.024	Easy: 0.219 ± 0.023	Easy: 0.060 ± 0.011	Easy: 0.217 ± 0.013	Easy: 0.161 ± 0.007	Easy: 0.124 ± 0.007
	Hard: 0.151 ± 0.028	Hard: 0.035 ± 0.004	Hard: 0.025 ± 0.003	Hard: 0.138 ± 0.007	Hard: 0.102 ± 0.003	Hard: 0.117 ± 0.010
Significance	*T*_49_ = 5.13,	*T*_49_ = 7.71,	*T*_49_ = 3.18,	*T*_49_ = 8.74,	*T*_49_ = 6.90,	*T*_49_ = −3.96,
	*p* < 0.0001	*p* < 0.0001	*p* = 0.003	*p* < 0.0001	*p* < 0.0001	*p* < 0.001

Significant differences according to the paired t test are shown in the last two rows.

### Data analyses

For each trial, we analyzed microsaccades occurring during three particular time windows (TWs) of 650 ms (see Fig. 1C). For TW 1 and TW 2, we set the time windows as 150 ms before the onsets of the cue and stimulus to 500 ms after them. Both monkeys’ bar release RTs were more than 150 ms; therefore, TW 3 was defined as the interval between 500 ms before and 150 ms after the target color change. The effects of attentional load on microsaccade parameters, including rate, amplitude and directional congruency, were quantified as follows.

### Time course of the microsaccade rate, amplitude and directional congruency

We respectively computed the microsaccade rate, amplitude and directional congruency in moving time windows, with a bin width of 50 ms and a step length of 10 ms, under different task difficulties within TW 1, 2 and 3. Then, we aligned each microsaccade’s amplitude and directional congruency in all trials within the corresponding time bin according to its onset time. To explore the time course of the microsaccade rate, we estimated the instantaneous rate in each time bin and computed its standard error of the mean (SEM) from five groups of data according to the locations of the target stimulus changing color. The changes of the microsaccade amplitude over time were assessed by calculating the mean values and their SEMs from all microsaccades aligned in each time bin. We studied the directional congruency between the microsaccade direction and the location of the color changing stimulus using a similar method proposed by Hafed *et al*.^38^. In each time bin, microsaccades with their direction within ±90° relative to the axis connecting the centers of the fixation cross and the color changing stimulus were assigned a value of 1, while microsaccades having the opposite directions were set as 0. The directional congruency ratio of 0.5 indicates that the fractions of microsaccades in both directions are equal. Thus, we studied the temporal dynamics of the microsaccade directional congruency by averaging all the assigned values in each time bin. Student t tests with a criterion of *p* < 0.05 were performed to test the significant differences in the microsaccade rate, amplitude and directional congruency in each time bin under the easy and hard conditions. To explore the difference in average microsaccade activities 500 ms after the onsets of cue and stimulus and 500 ms preceding the color change (Periods 1, 2 and 3 in Fig. 1) under different task difficulties, we computed the mean microsaccade rate, amplitude and directional congruency within 50 time bins and used ANOVA with a criterion of *p* < 0.05 for statistical analysis.

### Estimation of the microsaccade amplitude fluctuation

The fluctuation of the microsaccade amplitude may reflect the modulation of the perceptual load. The Fano factor provides a method to describe the variability in the collection of amplitudes and to relate it to the variability associated with a Poisson process. Let N_i_ for i = 1, …, n be a collection of independent identically distributed samples from a Poisson distribution. The Fano factor for these data is given by the ratio of the sample variance to the sample mean:

1 $${\rm{F}}=\,\frac{\frac{1}{{\rm{n}}-1}{\sum }_{{\rm{i}}=1}^{{\rm{n}}}{({{\rm{N}}}_{{\rm{i}}}-\bar{{\rm{N}}})}^{2}}{\bar{{\rm{N}}}},\,{\rm{w}}{\rm{h}}{\rm{e}}{\rm{r}}{\rm{e}}\,\bar{{\rm{N}}}=\frac{1}{{\rm{n}}}\sum _{{\rm{i}}=1}^{{\rm{n}}}{{\rm{N}}}_{{\rm{i}}}$$

We computed Fano factors of the microsaccade amplitude in 50 time bins of Periods 1, 2 and 3 for both task difficulties. A paired t test with a significance level of 0.05 was used to test the statistical significance of the differences in Fano factors under the easy and hard conditions during each period.

### The amount of task difficulty modulation index

The amount of the spatial attention and perceptual load effects on microsaccades was determined as a difficulty modulation index (DMI) according to the following equation:

2 $${\rm{D}}{\rm{M}}{\rm{I}}=\frac{{{\rm{M}}{\rm{S}}}_{{\rm{H}}}-{{\rm{M}}{\rm{S}}}_{{\rm{E}}}}{{{\rm{M}}{\rm{S}}}_{{\rm{H}}}+{{\rm{M}}{\rm{S}}}_{{\rm{E}}}}$$

where MS_H_ are the microsaccade activities, such as the rate, amplitude or directional congruency during the hard task, and MS_E_ are those during the easy task.

We computed the DMIs of the microsaccade activities (rate, amplitude, and directional congruency) in Periods 1, 2 and 3 and then used one-way ANOVA to compare them during different periods and a Bonferroni test for post hoc comparisons of each other. A positive index indicates that the microsaccade activities were enhanced during the hard task, whereas a negative index indicates that the microsaccade activities were suppressed.

### Relationship between microsaccades and behavioral performance

We measured both the RT and the correct rate as a function of time when a microsaccade was triggered relative to the onset of color change in both the easy and hard tasks. To acquire time courses of these effects, we aligned all the trials in which microsaccades occurred during the period between 200 ms before and 150 ms after the target color change and then used a running average with temporal bins (50 ms width) successively moved in 10 ms steps. We measured baselines of the RT and correct rate on all trials during which no microsaccades occurred within 350 ms around the time of color change. To detect the effect of microsaccades on behavioral performance, we compared with- and without-microsaccade situations within 350 ms around the target color change in easy and hard tasks from both monkeys (ANOVA with a criterion of *p* < 0.05).

All error bars and the ranges of error specified after the values of the mean were generated from SEM, i.e., mean ± SEM.

## Results

We analyzed behavioral data from 1458 easy task trials and 1379 hard task trials (collected from 23 easy and 23 hard blocks, respectively) performed by monkey G and 1435 easy task trials and 1339 hard task trials (collected from 24 easy blocks and 24 hard blocks) performed by monkey N.

### Task performance

For each trial, the reaction time (RT) was defined as the interval between the beginning of the target color change and the time of bar release. Errors resulting from fixation break were early release (the animal released the bar before the color change, or RT less than 150 ms) and late release (the animal did not respond, or RT more than 500 ms). We started analysis of the monkeys’ eye movements when they achieved better than 70% correct rates during the easy task. Overall, both monkeys responded more quickly and made fewer errors during the easy task. The overall RT and correct rate for each monkey were as follows (see Fig. 1A,B). For monkey G, the average RT was 284.5 ± 0.7 ms during the easy task and 346.6 ± 1.4 ms during the hard task (*F*_1,2835_ = 1709.6, *p* < 0.0001, one-way ANOVA). For monkey N, the average RT was 273.7 ± 1.0 ms during the easy task and 327.9 ± 1.8 ms during the hard task (*F*_1,2772_ = 707.7, *p* < 0.0001, one-way ANOVA). Monkey G achieved correct rates of 87.2 ± 2.4% during the easy task and 74.3 ± 2.1% during the hard task (*F*_1,44_ = 16.8, *p* < 0.001, one-way ANOVA). Monkey N made 84.3 ± 3.3% of correct actions during the easy task and 72.1 ± 3.8% during the hard task (*F*_1,46_ = 5.9, *p* = 0.02, one-way ANOVA). The animals became more impatient with increased task difficulty, since more fixation break errors occurred. All these behavioral data validate the efficiency of our perceptual load manipulation by changing the task difficulty.

Moreover, we cued a position that was different from the location where the grating changed color (10% of invalid cued trials) in several blocks. In these experiments, the RTs were significantly longer in the trials that were cued incorrectly (331.4 ± 1.8 ms vs. 292.7 ± 0.8 ms, *F*_1,255_ = 16.8, *p* < 0.001, one-way ANOVA), indicating that the animals were utilizing the cue to perform the task.

### Microsaccade rate

**Figure 2:**
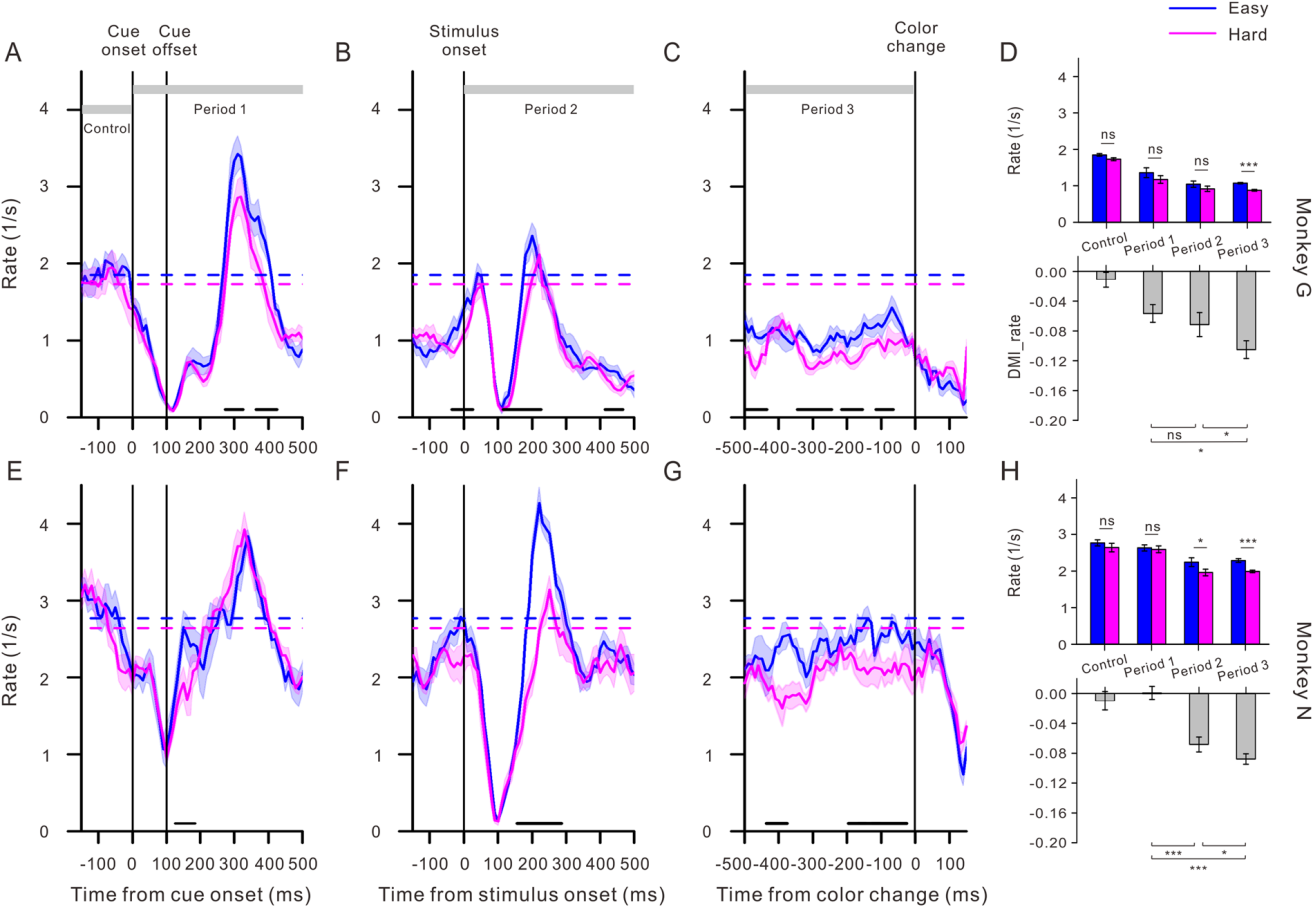
The temporal dynamics of the microsaccade rate and the modulations of perceptual load on the microsaccade rate during different time periods. (**A**–**C**,**E**–**G**) Temporal dynamics of the microsaccade rate during the easy (blue) and hard (pink) tasks for monkeys G (top panels) and N (bottom panels). The four vertical black lines indicate cue onset, cue offset, stimulus onset and target color change, respectively. The two horizontal dashed lines represent the baseline rate of microsaccades that occurred 150 ms before the cue onset (blue for the easy task and pink for the hard task). The gray lines at the top show the times of the Control and Periods 1, 2 and 3 in Fig. 1. The black lines at the bottom of each plot denote the periods in which the microsaccade rate under the two conditions differed significantly (*p* < 0.05, Student t test). (**D**,**H**) Comparisons of the perceptual load modulation on the microsaccade rate during different periods. For each monkey, the top panel shows the average microsaccade rate during the easy (blue) and hard (pink) tasks within different periods. The bottom panel shows the perceptual load modulation induced by task difficulty on the microsaccade rate (DMI). Error bars in all panels indicate SEM, and asterisks represent statistical significance (***p < 0.0001, **p < 0.001, *p < 0.05).

We first examined the microsaccade rate changes over time in three 650-ms time windows. Figure 2 shows the evolution of the microsaccade rate as a function of time across all trials in the easy and hard tasks for each monkey. The microsaccades had comparable baseline rates during the easy and hard tasks for monkey G (1.85 ± 0.04 Hz vs. 1.73 ± 0.04 Hz, *F*_1,28_ = 3.53, *p* = 0.06, one way ANOVA) and for monkey N (2.77 ± 0.08 Hz vs. 2.64 ± 0.12 Hz, *F*_1,28_ = 0.81, *p* = 0.38, one way ANOVA).

The temporal patterns of the microsaccade rate in relation to the cue onset/offset, stimulus onset, and color change were remarkably consistent. The microsaccade rate was relatively steady and was suppressed approximately 100–110 ms after the onsets of all stimulus events under both difficult conditions. The cue offset had a similar effect on the microsaccade rate, although this effect was weaker in monkey N. Approximately 200–250 ms after the cue offset and stimulus onset, the microsaccade rate rebounded in TW 1 and 2 for both monkeys, whereas there was no rebound in TW 3 due to the short time period between the color change and the monkey’s response. The microsaccade rate subsequently dropped to a lower level than the baselines before the color change. The microsaccade rate changes induced by the onsets of stimulus events are in agreement with those previously observed in humans and monkeys^6,10,24,28,39^. We further found that this microsaccade rate inhibition was not modulated by the perceptual load of the task.

On the other hand, a profound perceptual load modulation on the microsaccade rate during the rebound phase was found in TW 2 (Fig. 2B,F) but not TW 1. This significant microsaccade rate suppression by the perceptual load was also found immediately before the color change for both monkeys (Fig. 2C,G). We further analyzed the impact of the perceptual load on the microsaccade rate that occurred during the Control period (150 ms before the cue onset), Period 1 (500 ms after the cue onset), Period 2 (500 ms after the stimulus onset) and Period 3 (500 ms before the color change). As shown in the upper panels of Fig. 2D,H, high perceptual load significantly suppressed the microsaccade rate for both monkeys during Period 3 (1.07 ± 0.02 Hz vs. 0.88 ± 0.02 Hz, *F*_1,98_ = 45.27, *p* < 0.0001 for monkey G and 2.41 ± 0.03 Hz vs. 2.02 ± 0.03 Hz, *F*_1,98_ = 107.44, *p* < 0.0001 for monkey N, one way ANOVA). There were no significant difference during Period 1 (*F*_1,98_ = 1.17, *p* = 0.28 for monkey G and *F*_1,98_ = 0.05, *p* = 0.83 for monkey N, one way ANOVA). In addition, there were no consistent difference during Period 2 (1.05 ± 0.09 Hz vs. 0.92 ± 0.08 Hz, *F*_1,98_ = 1.25, *p* = 0.27 for monkey G and 2.22 ± 0.14 Hz vs. 1.90 ± 0.10 Hz, *F*_1,98_ = 4.49, *p* = 0.05 for monkey N, one way ANOVA).

To quantify the perceptual load modulation on the microsaccade rate during the four periods, we calculated the DMI_rate ratios (see methods). The DMI_rate was close to zero during the Control period, showing no modulation of the perceptual load, since there was no peripheral stimulus present. Values of the DMI_rate were less than zero during Periods 2 and 3 for both monkeys, indicating an inhibition of the microsaccade rate by the perceptual load. The analysis revealed a significant effect of time period on the DMI_rate (*F*_3,161_ = 3.53, *p* = 0.02 for monkey G and *F*_3,161_ = 16.72, *p* < 0.0001 for monkey N, one way ANOVA). The Bonferroni post hoc comparisons revealed significantly greater DMI_rate during the Period 3 compared to the Period 1 (−0.10 ± 0.01 vs. −0.06 ± 0.01, *p* = 0.01 for monkey G and −0.09 ± 0.01 vs. −0.0007 ± 0.01, *p* < 0.0001 for monkey N) and the Period 2 (−0.10 ± 0.01 vs. −0.07 ± 0.02, *p* = 0.05 for monkey G and −0.09 ± 0.01 vs. −0.07 ± 0.01, *p* = 0.05 for monkey N), indicating that the strongest modulation effect of the perceptual load on the microsaccade rate appeared immediately before the occurrence of the response event.

In summary, even though the microsaccade rate was suppressed during the rebound phase after the stimulus onset, the sustained and stronger modulation of the perceptual load on them appeared before the target color change.

### Microsaccade amplitude

Generally, both monkeys made small microsaccades even before the cue onset while performing the demanding detection task. For monkey G, the mean amplitudes were 0.23° ± 0.015° and 0.22° ± 0.010°(*F*_1,28_ = 0.16, *p* = 0.69, one way ANOVA) in the Control period during the easy task and hard task, respectively. For monkey N, the mean amplitudes were 0.21° ± 0.006°in the easy task and 0.21° ± 0.006° in the hard task (*F*_1, 28_ = 0.53, *p* = 0.47, one way ANOVA).

**Figure 3:**
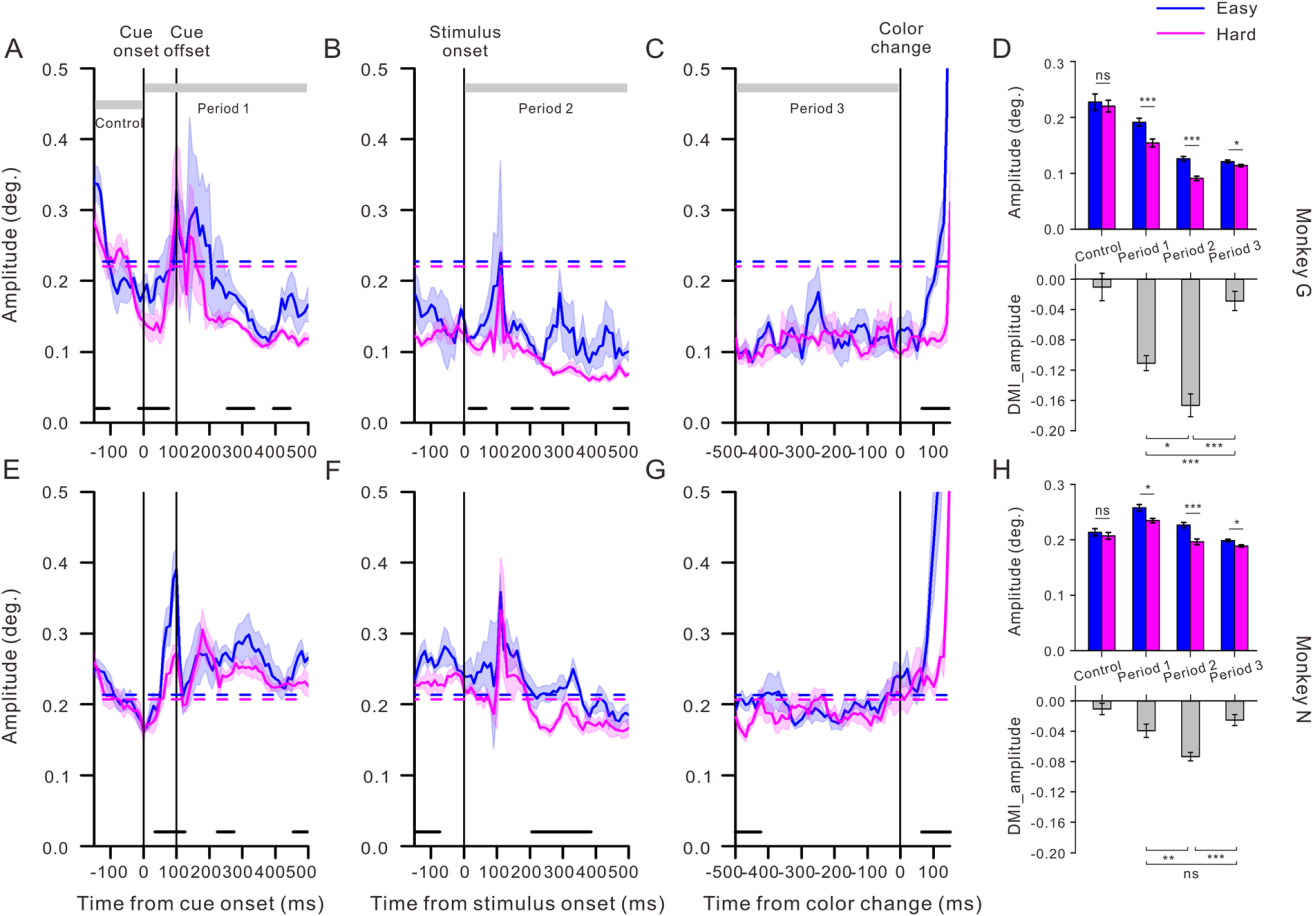
The temporal dynamics of the microsaccade amplitude and the modulations of perceptual load on the microsaccade amplitude during different time periods. (**A**–**C**,**E**–**G**) Temporal comparison of the microsaccade amplitude between the easy (blue) and hard (pink) conditions for monkeys G and N. For each monkey, the microsaccade amplitude demonstrated a transient increase around the time of the minimum rate seen in the earlier figure after the cue or stimulus appearance and the target color change onset. The horizontal dashed lines represent the average amplitude during the period of Control. The black lines at the bottom of each plot demonstrate the periods in which the microsaccade amplitude under the two conditions differed significantly (*p* < 0.05, Student t test). The gray rectangles are defined as in Fig. 2. (**D**,**H**) Measures of the perceptual load modulation on the microsaccade amplitude during different periods. For both monkeys, the top panels show the average microsaccade amplitude during the easy (blue) and hard (pink) tasks in the four periods. The bottom panels show the DMI ratios of the microsaccade amplitude within the four time periods. Error bars indicate SEMs, which are defined in Fig. 2.

To explore a possible interaction between the perceptual load and the microsaccade amplitude, we performed an analysis similar to the one we performed on the microsaccade rate. In Fig. 3, the microsaccade amplitude demonstrated a transient increase of 100–110 ms when a stimulus event occurred, exactly at the time of the minimum microsaccade rate. When the microsaccade amplitude increased after the cue onset, an inconsistent effect of the perceptual load on the microsaccade amplitude has been found for the two monkeys (see Fig. 3AE).

To evaluate the effect of the perceptual load modulation on the microsaccade amplitude, we calculated mean amplitudes across all trials and corresponding DMI ratios during each time period under both task difficulties. Here, we observed a suppressing effect of the perceptual load on the microsaccade amplitude for both monkeys. The suppressing effect was statistically significant during all periods after the cue onset, as follows: Period 1, 0.19° ± 0.007° vs. 0.15° ± 0.007°, *F*_1,98_ = 13.51, *p* < 0.0001 for monkey G and 0.26° ± 0.006° vs. 0.23° ± 0.004°, *F*_1, 98_ = 10.48, *p* = 0.002 for monkey N, Period 2, 0.13° ± 0.004° vs. 0.09° ± 0.004°, *F*_1,98_ = 37.20, *p* < 0.0001 for monkey G and 0.23° ± 0.005° vs. 0.20° ± 0.005°, *F*_1,98_ = 19.27, *p* < 0.0001 for monkey N, and Period 3, 0.12 ± 0.003° vs. 0.11 ± 0.002°, *F*_1,98_ = 4.66, *p* = 0.03 for monkey G and 0.20 ± 0.002° vs. 0.19 ± 0.002°, *F*_1,98_ = 10.39, *p* = 0.002 for monkey N (easy vs. hard, one way ANOVA). There was also a significant effect of time period on the DMI_amplitude (*F*_3,161_ = 24.88, *p* < 0.0001 for monkey G and *F*_3,161_ = 10.63, *p* < 0.0001 for monkey N). As shown at the bottom panels of Fig. 3D,H, the absolute values of the DMI_amplitude were the greatest in Period 2 for both monkeys, suggesting that the strongest modulation by the perceptual load on the microsaccade amplitude occurred after the stimulus onset.

Furthermore, a lower fluctuation of amplitudes during the hard task after the cue onset was found. Table 1 presents a comparison of the Fano factors of the microsaccade amplitudes during the easy and hard tasks. The Fano factors always exhibited a significant difference during Periods 1, 2 and 3 in both monkeys, indicating lower fluctuations of microsaccade amplitude under the high perceptual load.

Taken together, these observations revealed that the microsaccade amplitude and its fluctuation were suppressed by the perceptual load immediately after the cue onset, and the strongest effect emerged after the stimulus onset.

### Directional congruency

**Figure 4:**
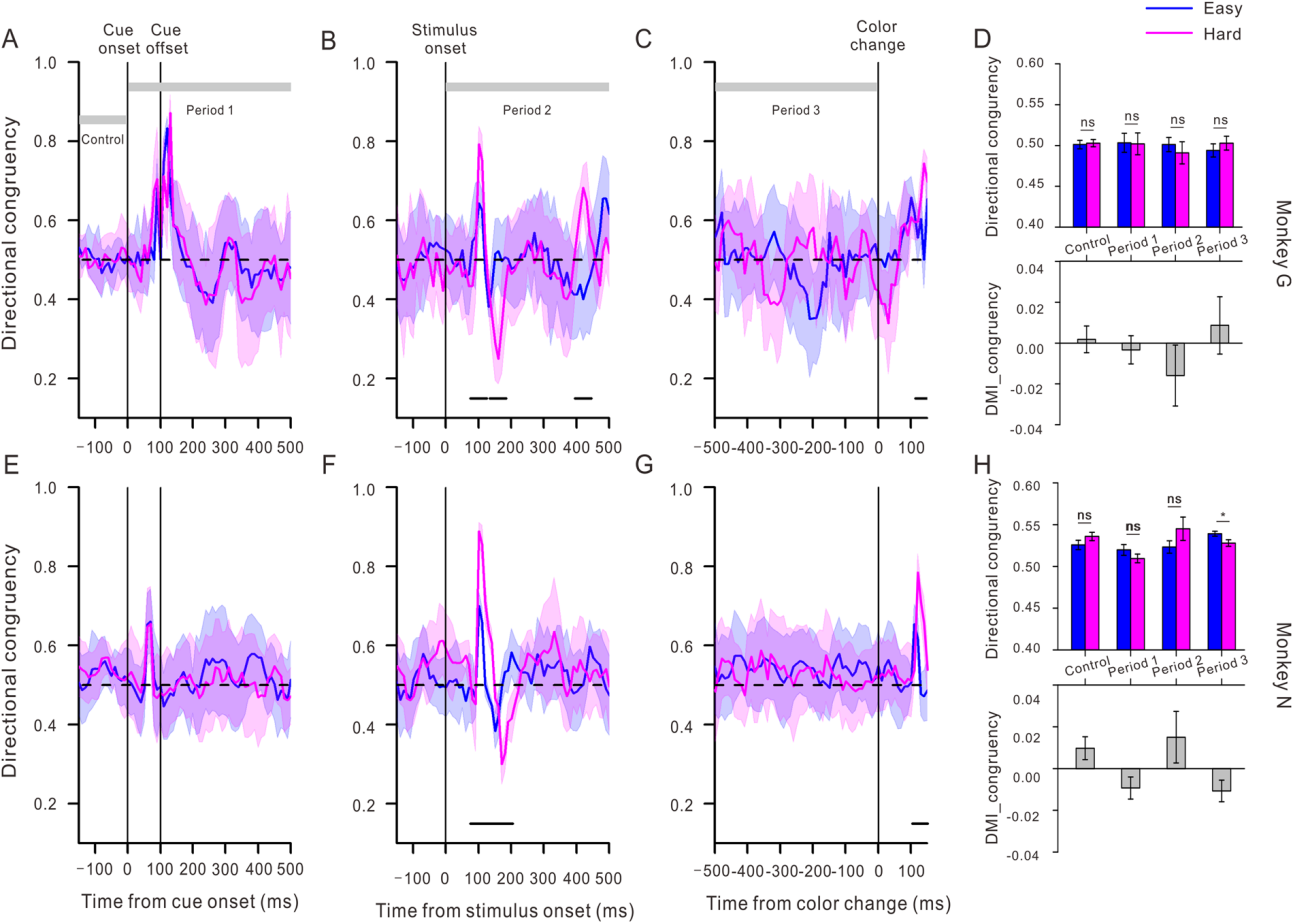
The temporal dynamics of the microsaccade directional congruency and the modulations of perceptual load on the microsaccade directional congruency during different time periods. (**A**–**C**,**E**–**G**) Temporal comparison of the directional congruency ratios between the easy (blue) and hard (pink) tasks for monkeys G and N. The horizontal black dashed lines represent a *y*-axis value of 0.5 and indicate no microsaccade directional bias toward the indicated location. The black lines at the bottom of each plot denote the periods in which the directional congruency from the easy and hard tasks differed significantly (*p* < 0.05, Student t test). The gray rectangles are defined as in earlier figures. (**D**,**H**) Measures of the perceptual load modulation on the overall congruency of the microsaccade direction during the four periods. The top panels depict the average directional congruency in the easy (blue) and hard (pink) tasks during the four periods. The bottom panels demonstrate the DMI ratios of the direction congruency within the four time periods. The figure conventions are the same as the previous figures.

We also explored possible interaction between the perceptual load and the fraction of microsaccades biased by the peripheral cue. Figure 4 shows that the microsaccade direction exhibited a strong early bias around 100 ms toward the peripheral location with a stimulus event, similar to the time of change of the microsaccade rate and amplitude. This directional bias towards the peripheral stimulus may be driven by attention mechanisms devoted to gaining information about the target. However, the directional congruency of microsaccades generally does not deviate from the ratio of 0.5 on the other time bins, suggesting a strategy of directing back and forth between the cued and opposite locations at approximately equal rates. Even though the directional congruency of microsaccades occurring around 100 ms after stimuli onset and target color change significantly differed between the easy and hard conditions, indicating a transient effect of the perceptual load (see Fig. 4B,C,F,G), it was nonetheless modulated by the perceptual load within almost all the time periods (note that the overall directional congruency ratio in the easy task was significantly larger than that in the hard task during Period 4 only for monkey N; see Fig. 4H, top panels). This was confirmed by the DMI ratios of the directional congruency. The absolute values of the DMI in the four periods were close to zero, showing no modulation of the perceptual load in all the time periods (*F*_3,161_ = 0.72, *p* = 0.54, one way ANOVA).

### Effect of microsaccades on behavioral performance

We also analyzed the influence of microsaccades that occurred around the onset of the color change on behavioral performance. For comparison to microsaccade-free situations, we measured the baseline RT and correct percentage on all trials during which no microsaccades occurred within 350 ms of the target color change onset. For monkey G, the no-microsaccade baselines of the RT were 282.5 ± 0.74 ms in the easy task and 343.4 ± 1.5 ms in the hard task, and they were 276.5 ± 1.2 ms and 332.9 ± 2.1 ms for monkey N. The no-microsaccade baselines of the correct percentage were 89.8 ± 2.3% for the easy task and 75.7 ± 2.2% for the hard task for monkey G and 84.3 ± 2.4% and 72.4 ± 3.4% for monkey N.

**Figure 5:**
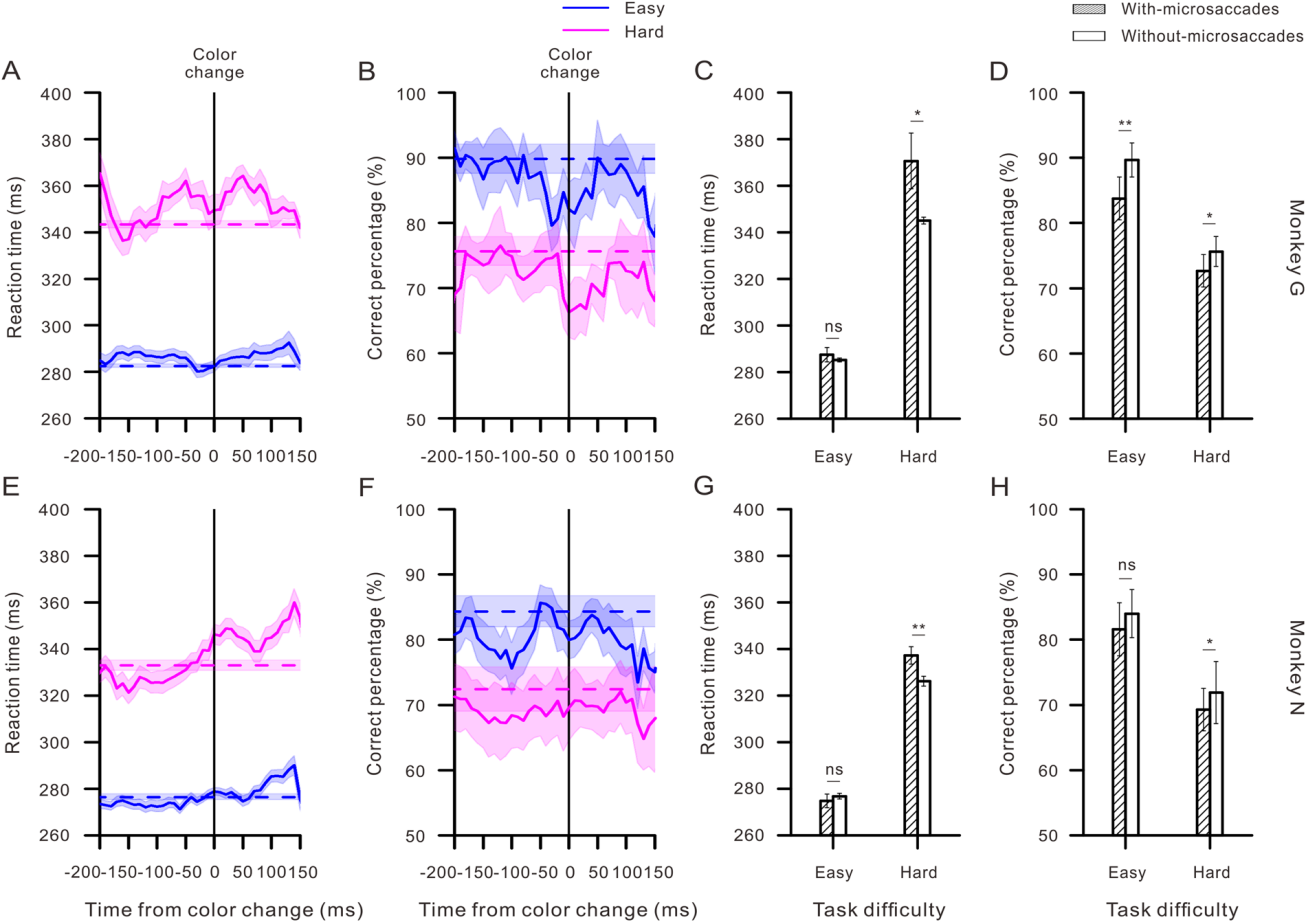
Monkeys’ performances when microsaccades occurred around the target color change under the easy and hard conditions. (**A**,**E**) The RT as a function of when microsaccades occurred around the color change onset. The vertical lines indicate the occurrence of a target color change. The horizontal dashed lines represent the average RT of all trials in which there were no microsaccades occurring within 350 ms around the target color change. There was a tendency toward longer RTs when microsaccades occurred during this period. (**B**,**F**) Same as in **A**, **E** but for the correct percentage. In both monkeys, microsaccades occurring around the response event were correlated with a decrease in accuracy. (**C**,**G**) Comparisons of the overall RT between the with- and without-microsaccade situations in both easy and hard tasks from the two monkeys. (**D**,**H**) Same as in **C**, **G** but for the overall accuracy. Error bars indicate SEM, and asterisks represent statistical significance (***p < 0.0001, **p < 0.001, *p < 0.05).

The temporal patterns of the RT and correct rate associated with the microsaccades occurring relative to the target color change are shown in Fig. 5. As depicted in Fig. 5A,E, both monkeys exhibited longer RT when microsaccades occurred around the target color change. We then compared these reaction times in the easy and hard tasks with those observed on the microsaccade-free trials. As shown in Fig. 5C,G, the reaction times of trials with microsaccades were significantly longer than those without microsaccades during the hard task (monkey G, 370.6 ± 12.0 ms vs. 345.0 ± 1.5 ms, *F*_1,1212_ = 4.57, *p* = 0.03, monkey N, 337.3 ± 3.8 ms vs. 326.2 ± 2.1 ms, *F*_1,1207_ = 13.54, *p* < 0.001, one way ANOVA).

Microsaccades occurring near the response event were also associated with changes in the monkeys’ accurate percentages. We performed an analysis on the accurate rate (see Fig. 5B,F) and found a manifest tendency toward lower accuracy in the cases with microsaccades than those without microsaccades, especially during the hard tasks (see Fig. 5D,H). For monkey G, the mean accurate percentages were 83.8 ± 3.3% vs. 89.7 ± 2.6%, *F*_1,1417_ = 13.58, *p* < 0.001 and 72.6 ± 2.3% vs. 75.6 ± 2.3%, *F*_1,1212_ = 4.35, *p* = 0.04 in the easy and hard tasks, respectively. For monkey N, they were 81.6 ± 4.1% vs. 84.0 ± 3.7%, *F*_1,136_ = 3.23, *p* = 0.07 in the easy task and 69.3 ± 3.3% vs. 71.9 ± 4.8%, *F*_1,1207_ = 3.88, *p* = 0.04 in the hard task (with vs. without microsaccades, one way ANOVA).

Generally speaking, microsaccades impaired the monkeys’ behavioral performance, especially in the hard tasks with high perceptual load. The increase in the RT and the decrease in the accuracy are likely a manifestation of microsaccade suppression.

## Discussion

The majority of previous work studying attentional load effects on microsaccades used complex tasks involving the working memory load or perceptual load in human subjects. However, the present study focused on microsaccade behaviors while performing a task primarily with visual perceptual processing in monkeys. We manipulated the perceptual load level by task difficulty within alternate easy and hard blocks. After assessing the temporal effect of the perceptual load on the microsaccade rate, amplitude and directional congruency, we found that the modulation may vary across time. Our results show that a higher perceptual load was associated with the generation of fewer microsaccades and with smaller and more-stable amplitudes.

### Effects of stimulus onset on microsaccadic activities

Consistent with studies that have shown that microsaccades are affected by any stimulus transients^10,33,38–40^, we also observed a fast initial decrease in the microsaccade rate with an abrupt onset or offset of the surrounding patterns under all conditions. Moreover, we found that the microsaccade amplitude demonstrated a transient increase exactly at the time of the minimum microsaccade rate. Specially, the fact that task difficulty had no effect on the initial inhibition supports the notion that this inhibition of microsaccades after the stimulus transients is a fast reflex of the oculomotor system, that is, it is bottom-up or stimulus driven^10,33^. Following the inhibition period, the microsaccade rate increased and presented a rebound that was strongly modulated by the perceptual load. Meanwhile, it should be noted that the microsaccade directions around the inhibition episode were clearly oriented to the spatial location of attention focus and then to the opposite direction, like earlier studies in humans^6^ and monkeys^38^. This phenomenon was more evident during the hard task (Fig. 4B,F). These results suggest that although the inhibition phase of microsaccades induced by the stimulus transients is stimulus driven, its rebound phase with a long latency may be influenced by voluntary or top-down control^33^.

### The influence of microsaccades at the time of color change on behavioral performance

Even though many studies concerning microsaccades focus on their roles report that they triggered excitatory neural responses in most brain areas^4,41,42^, and agree that they generally benefit vision^41,43–46^, microsaccades have also been shown to suppress neural activity^47,48^ and inhibit behavioral performance^10^ during stimulus presentation in monkeys. Our results revealed a detrimental effect of microsaccades on the task performance as well. Microsaccades appeared before the target color change seemed to decrease the speed and efficiency of the behavioral response, especially in the hard tasks with high perceptual load, suggesting that the inhibition of microsaccade generation might aid the detection of target change.

Although our results clearly demonstrate the influence of microsaccades on perceptual performance, a potential contribution from attentional allocation to this effect must be carefully evaluated. For example, it could be argued that more microsaccades could result from inattentiveness, which in turn could have lowered perceptual performance. There are several reasons why attentional allocation is unlikely to affect our measurements. First, the color change was very subtle even in the easy task, therefore the monkeys would perform the tasks with great effort. Second, we observed that the monkeys executed saccades to the target location after response in most trials, indicating they were paying attention to the perceptual target. Finally, since the monkeys were primarily driven by the magnitude of reward (the faster the response, the larger the reward), it is unlikely that they would decrease their effort during the easy trials.

### Effects of attentional load on microsaccade activities

The load theory has argued that perceptual load and working memory load produce opposite effects on irrelevant interference. Hence, they need to be separated from each other in order to rule out possible bias to either one. In previous studies which investigated the effects of attentional load on the microsaccade rate, however, both the visual processing and working memory could have contributed to the microsaccadic modulation. In those nonvisual tasks, participants were required to maintain fixation and perform mental operations, such as mental counting^25,26^, arithmetic operation^27,28^ or memory retention^29^. In the visual task of identifying letter color vs. letter shape^9^, the presentation of mask between stimulus display and response resulted in working memory demands since subjects needed to remember the target color or shape. In the dual task of simulated driving experiment^23^, participants were asked to perform visual search, which required representing the target template, comparing the target template to distractors, and categorizing the stimulus items in the search array. Accordingly, visual working memory may play a critical role in this searching process. Participants in the experiment administrated by Hicheur *et al*.^24^ had to confirm their first judgment at the end of stimulus presentation, consequently they would memorize the judgment until the second response. In these regards, there could be complicated interactions between the effects of the perceptual and the working memory load in the aforementioned tasks, which caused inconsistent effects of the attentional load on the microsacadic activities. In our study, the monkeys had no need to memorize any visual or spatial information while conducting the demanding detection task. Thus, we are able to examine the dynamic changes of microsaccade response in different conditions of perceptual load with limited involvement of working memory.

We found the microsaccade rate was significantly lower with high load than with low load in both monkeys. This was consistent with those nonvisual studies associated with working memory^9,25,27–29^ and the visual discrimination task^9^ in humans. In contrast, some other studies reported an opposite effect of the attentional load on the microsaccade rate^23,24^. For those nonvisual tasks, mental cognitive demands could be primary, whereas the visual demands could be secondary due to their little effects on task performance. Microsaccades were neither essential nor beneficial for completing the primary task, which could explain the suppressive effect of working memory load reported by these studies. For visual tasks, we proposed that the effect of attentional load on microsaccade rate might depend on whether microsaccades have perceptual cost or benefit in a specific situation. In our study, we found a detrimental effect of microsaccades on the speed and efficiency of the animals’ behavioral response. For this reason, it is likely that suppressing microsaccades may be advantageous for the peripheral visual detection^4,10^. On the contrary, Benedetto *et al*.^23^ argued that the increment of free-view microsaccades in the dual-task condition consisting of simulated driving and visual searching was helpful to gain a more frequent sampling for visual exploration. Hicheur *et al*.^24^ asked participants to fixate on the target, therefore the enhancement of microsaccade rate during the execution of visual discrimination may be devoted to gain spatial information about the target orientation. In summary, the perceptual costs or benefits of microsaccades might drive the observers to voluntarily adjust the fixation strategy for facilitating their behavioral performance.

Furthermore, we assessed the perceptual load effect at a fine temporal scale to observe possible fluctuations in the microsaccade behaviors. We found that the modulation of the microsaccade rate by the perceptual load was significantly stronger during the rebound phase of the microsaccade rate and the period before the target color change. Different time periods within the task may cause different cognitive requirements to accommodate visual demand. The dynamics of modulation on the microsaccade rate could be related to the temporal fluctuations in the perceptual load. Specially, the monkeys in our study had to maintain attention for detecting the upcoming color change and simultaneously keep a precise fixation until their response. Therefore, the effect of attentional load on the microsaccade rate could last for a long time.

The microsaccade amplitude was also compared between time periods within trials. Although the average microsaccade amplitude was significantly inhibited by higher perceptual load, the strongest modulation occurred after the stimulus onset rather than before the target color change. The weaker modulation on the amplitude prior to the color change may have been caused by the highly suppressed microsaccade amplitude during both the easy and hard tasks. As we mentioned above, microsaccades in our task might have possible detrimental effects of microsaccades around the target color change. Therefore, the more difficult the task, the stronger the monkeys would control their amplitudes of microsaccades. However, two other studies^24,27^ have reported different effects of the attentional load on the microsaccade amplitude. Siegenthaler *et al*.^27^ detected microsaccades in a nonvisual arithmetic task and found that their amplitudes increased with higher task difficulty. In their tasks, it was reasonable to assume that the participants might allocate their attention primarily to mental counting and secondarily to fixation maintaining. Therefore, when working memory load was higher, the participants may not maintain precise fixation and produce larger microsaccades due to the weaker control of their oculomotor system. Hicheur *et al*.^24^ found stimulus-driven directional bias, which was advantageous for behavioral performance, was observed for both small (0.1°–0.5°) and large (0.5°–1°) microsaccades. Hence, there was no significant difference of microsaccade amplitude between the conditions of high and low attentional load. Generally, these discrepancies were presumably caused by the complicated interaction of perceptual and working memory load, and the effect of microsaccades on observers’ behavior performance. Besides, Chen *et al*.^30^ had previously conducted an analogous experiment and detected microsaccades within 500 ms before the response event. They found no significant modulation of task difficulty on the microsaccade amplitude in this period. It might be that the primates were highly motivated before the response event onset under both difficulties, which in turn would suppress the amplitude such that it was nearly the same in both the easy and hard tasks.

### Plausible mechanisms for the effects of perceptual load on microsaccades

The superior colliculus (SC) is traditionally divided into two areas, the rostral fixation zone and the caudal saccade zone, with neurons that are activated respectively during microsaccades and saccades^49^. Previous electrophysiological research^50^ has found that fluctuations in SC activity at the rostral pole play a causal role in microsaccade generation during fixation. Furthermore, the rostral SC receives excitatory inputs from the frontal and parietal areas and inhibitory inputs from the basal ganglia. Given that these cortical areas are involved in visual attention^51^, varying the perceptual load should modulate rostral SC activity and, thus, the microsaccade rate and amplitude.

In conclusion, we assessed the effectiveness of two perceptual load levels to modulate microsaccades in monkeys. We demonstrated that high perceptual load suppressed the rate and amplitude of microsaccades, suggesting that these two parameters could be indicators of the perceptual load.

## References

[1] Martinez-Conde S, Macknik SL, Hubel DH (2000). Microsaccadic eye movements and firing of single cells in the striate cortex of macaque monkeys. Nat. Neurosci..

[2] Rolfs M (2009). Microsaccades: small steps on a long way. Vision Res..

[3] Martinez-Conde S, Macknik SL, Troncoso XG, Hubel DH (2009). Microsaccades: a neurophysiological analysis. Trends Neurosci..

[4] Martinez-Conde S, Otero-Millan J, Macknik SL (2013). The impact of microsaccades on vision: towards a unified theory of saccadic function. Nat. Rev. Neurosci..

[5] Otero-Millan J, Macknik SL, Langston RE, Martinez-Conde S (2013). An oculomotor continuum from exploration to fixation. Proc. Natl. Acad. Sci. USA.

[6] Engbert R, Kliegl R (2003). Microsaccades uncover the orientation of covert attention. Vision Res..

[7] Laubrock J, Engbert R, Kliegl R (2005). Microsaccade dynamics during covert attention. Vision Res..

[8] Hafed ZM, Clark JJ (2002). Microsaccades as an overt measure of covert attention shifts. Vision Res..

[9] Pastukhov A, Braun J (2010). Rare but precious: microsaccades are highly informative about attentional allocation. Vision Res..

[10] Hafed ZM, Lovejoy LP, Krauzlis RJ (2011). Modulation of microsaccades in monkey during a covert visual attention task. J. Neurosci..

[11] Galfano G, Betta E, Turatto M (2004). Inhibition of return in microsaccades. Exp. Brain. Res..

[12] Rolfs M, Engbert R, Kliegl R (2005). Crossmodal coupling of oculomotor control and spatial attention in vision and audition. Exp. Brain. Res..

[13] Yuval-Greenberg S, Merriam EP, Heeger DJ (2014). Spontaneous microsaccades reflect shifts in covert attention. J. Neurosci..

[14] Laubrock J, Kliegl R, Rolfs M, Engbert R (2010). When do microsaccades follow spatial attention?. Atten. Percept. Psychophys..

[15] Lavie N, Tsal Y (1994). Perceptual load as a major determinant of the locus of selection in visual Atten. Percept. Psychophys..

[16] Urbach D, Spitzer H (1995). Attentional effort modulated by task difficulty. Vision Res..

[17] Lavie N, Hirst A, de Fockert JW, Viding E (2004). Load theory of selective attention and cognitive control. J. Exp. Psychol. Gen..

[18] Lavie N (2005). Distracted and confused? selective attention under load. Trends Cogn. Sci..

[19] Lavie N (2010). Attention, distraction, and cognitive control under load. Current Directions in Psychological Science.

[20] Schwartz S (2005). Attentional load and sensory competition in human vision: modulation of fMRI responses by load at fixation during task-irrelevant stimulation in the peripheral visual field. Cereb. Cortex.

[21] Rissman J, Gazzaley A, D’Esposito M (2009). The effect of non-visual working memory load on top-down modulation of visual processing. Neuropsychologia.

[22] Kelley TA, Lavie N (2011). Working memory load modulates distractor competition in primary visual cortex. Cereb. Cortex.

[23] Benedetto, S., Pedrotti, M. & Bridgeman, B. Microsaccades and exploratory saccades in a naturalistic environment. *J*. *Eye Mov*. *Res*. **4** (2011).

[24] Hicheur H, Zozor S, Campagne A, Chauvin A (2013). Microsaccades are modulated by both attentional demands of a visual discrimination task and background noise. J. Vis..

[25] Valsecchi M, Betta E, Turatto M (2007). Visual oddballs induce prolonged microsaccadic inhibition. Exp. Brain Res..

[26] Valsecchi M, Turatto M (2009). Microsaccadic responses in a bimodal oddball task. Psychol. Res..

[27] Siegenthaler E (2014). Task difficulty in mental arithmetic affects microsaccadic rates and magnitudes. Eur. J. Neurosci..

[28] Gao, X., Yan, H. & Sun, H. J. Modulation of microsaccade rate by task difficulty revealed through between- and within-trial comparisons. *J*. *Vis*. **15** (2015). 10.1167/15.3.3 25740876

[29] Dalmaso M, Castelli L, Scatturin P, Galfano G (2017). Working memory load modulates microsaccadic rate. J. Vis..

[30] Chen Y (2008). Task difficulty modulates the activity of specific neuronal populations in primary visual cortex. Nat. Neurosci..

[31] Judge SJ, Richmond BJ, Chu FC (1980). Implantation of magnetic search coils for measurement of eye position: an improved method. Vision Res..

[32] Engbert R, Mergenthaler K (2006). Microsaccades are triggered by low retinal image slip. Proc. Natl. Acad. Sci. USA.

[33] Engbert R (2006). Microsaccades: a microcosm for research on oculomotor control, attention, and visual perception. Prog. Brain Res..

[34] McCamy MB (2012). Microsaccadic efficacy and contribution to foveal and peripheral vision. J. Neurosci..

[35] Moller F, Laursen ML, Tygesen J, Sjolie AK (2002). Binocular quantification and characterization of microsaccades. Graefes. Arch. Clin. Exp. Ophthalmol..

[36] Zuber BL, Stark L, cook G (1965). Microsaccades and the velocity–amplitude relationship for saccadic eye movements. Science.

[37] Martinez-Conde S, Macknik SL, Hubel DH (2004). The role of fixational eye movements in visual perception. Nat. Rev. Neurosci..

[38] Hafed ZM, Ignashchenkova A (2013). On the dissociation between microsaccade rate and direction after peripheral cues: microsaccadic inhibition revisited. J. Neurosci..

[39] Engbert R (2012). Computational modeling of collicular integration of perceptual responses and attention in microsaccades. J. Neurosci..

[40] Rolfs M, Kliegl R, Engbert R (2008). Toward a model of microsaccade generation: the case of microsaccadic inhibition. J. Vis..

[41] Martinez-Conde S, Macknik SL, Hubel DH (2000). Microsaccadic eye movements and firing of single cells in the striate cortex of macaque monkeys. Nat. Neurosci..

[42] Troncoso XG (2015). V1 neurons respond differently to object motion versus motion from eye movements. Nat. commun..

[43] Ko HK, Poletti M, Rucci M (2010). Microsaccades precisely relocate gaze in a high visual acuity task. Nat. Neurosci..

[44] Chen CY, Ignashchenkova A, Thier P, Hafed ZM (2015). Neuronal Response Gain Enhancement prior to Microsaccades. Curr. Biol..

[45] Martinez-Conde S, Macknik SL, Troncoso XG, Dyar TA (2006). Microsaccades counteract visual fading during fixation. Neuron.

[46] McCamy MB, Otero-Millan J, Di Stasi LL, Macknik SL, Martinez-Conde S (2014). Highly Informative Natural Scene Regions Increase Microsaccade Production during Visual Scanning. J. Neurosci..

[47] Hafed ZM, Krauzlis RJ (2010). Microsaccadic suppression of visual bursts in the primate superior colliculus. J. Neurosci..

[48] Herrington TM (2009). The effect of microsaccades on the correlation between neural activity and behavior in middle temporal, ventral intraparietal, and lateral intraparietal areas. J. Neurosci..

[49] Munoz DP, Wurtz RH (1993). Fixation cells in monkey superior colliculus. I. Characteristics of cell discharge. J. Neurophysiol..

[50] Hafed ZM, Goffart L, Krauzlis RJ (2009). A neural mechanism for microsaccade generation in the primate superior colliculus. Science.

[51] Schall JD (2004). On the role of frontal eye field in guiding attention and saccades. Vision Res..

